# Hypertension and blood lead levels among battery technicians and office workers in western Nigeria

**DOI:** 10.4102/jphia.v17i1.1825

**Published:** 2026-09-23

**Authors:** Ebenezer A. Ozomata, Blossom A. Maduafokwa, Ugonnaya U. Igwilo, Emmanuel F. Osagiede, Uzoamaka A. Igwilo, Opeyemi Giwa

**Affiliations:** 1 Department of Community Medicine, Faculty of Clinical Sciences, College of Medicine and Health Sciences, Baze University, Abuja, Nigeria; 2 Department of Health Policy, Tecon Oil Services Ltd, Lagos, Nigeria; 3 Department of Community Health, Faculty of Public Health, National Postgraduate Medical College of Nigeria, Ijanikin, Nigeria; 4 Department of Clinical Science Research, University of East Anglia, Norwich, United Kingdom; 5 Department of Community Health, Faculty of Health Services, Eko Electricity Distribution Company, Lagos, Nigeria; 6 Department of Health Services, Lagos State Health Services Commission, Lagos, Nigeria

**Keywords:** battery technicians, office workers, hypertension, risk factors, occupational exposure, lead levels, Nigeria

## Abstract

**Background:**

Occupational exposure to lead remains a significant public health concern because of its association with hypertension and cardiovascular disease. Battery technicians are particularly at risk because of prolonged exposure to lead-containing materials.

**Aim:**

This study assessed and compared blood lead levels (BLL), blood pressure (BP) profiles and associated risk factors among battery technicians and office workers in Western Nigeria.

**Setting:**

The study was conducted in Lagos State, Nigeria.

**Methods:**

A cross-sectional study design was employed using multistage sampling. Data were collected with a structured interviewer-administered questionnaire, and BP measurements were obtained. Data analysis was performed using the Statistical Package for Social Sciences version 20. Chi-square, Mann–Whitney U, Fisher’s exact and Student’s *t*-tests were applied at a significance level of *p* ≤ 0.05.

**Results:**

Median systolic blood pressure (SBP) was statistically significantly higher among battery technicians (124 mmHg) compared to office workers (119.33 mmHg) (*p* = 0.029). However, the prevalence of elevated SBP (> 140 mmHg) was 22% in technicians and 18% in office workers. Among technicians, age and years of work were significantly associated with both SBP and diastolic blood pressure. While 30% of technicians had BLL ≥ 5.00 µg/dL, all office workers had levels below this threshold (*p* < 0.001). Multivariable logistic regression demonstrated that elevated BP was independently driven by advancing age (adjusted odds ratio [aOR]: 3.92; 95% confidence interval [CI]: 1.52–10.12; *p* = 0.005), occupational longevity exceeding 10 years (aOR: 2.54; 95% CI: 1.18–5.46; *p* = 0.017) and work intensity over 8 h daily (aOR: 2.12; 95% CI: 1.09–4.13; *p* = 0.027).

**Conclusion:**

Battery technicians exhibited higher SBP levels than office workers. Improved workplace safety practices, regular use of protective devices, personal hygiene and routine biological monitoring may reduce elevated lead and hypertension among battery technicians in Nigeria.

**Contribution:**

Improved workplace safety and practices, regular use of PPEs and personal hygiene, and routine biological monitoring among battery technicians in Nigeria may reduce elevated lead and hypertension among battery technicians.

## Introduction

Occupational lead exposure remains a pervasive global health challenge, with battery technicians being particularly vulnerable because of lead fume inhalation, oral ingestion and dermal penetration. These exposure routes facilitate lead’s entry into the bloodstream, triggering multi-systemic toxicity, most notably within the cardiovascular system.^1^ The World Health Organization has identified lead-induced hypertension as a significant occupational risk factor for cardiovascular disease, yet the strength of this association appears to vary across different geographic and industrial contexts.^2,3^

Globally, research has produced divergent findings regarding the link between lead and blood pressure (BP). Studies in industrialised nations such as Italy, Taiwan and South Korea have reported strong correlations between cumulative lead exposure and elevated systolic blood pressure (SBP).^4,5,6,7^ Conversely, data from developing regions show less consistency; while studies in Bangladesh and India mirror the findings of industrialised nations, research in Iran and Pakistan found no significant association between blood lead levels (BLL) and hypertension.^1,8,9,10^ These discrepancies suggest that the cardiovascular impact of lead may be modulated by regional factors, such as smoking prevalence, dietary habits and the specific nature of the work environment.

In Nigeria, the battery repair industry operates primarily within the informal sector, characterised by roadside workshops with minimal health oversight and high vehicular density.^11^ Despite the high number of young men employed in this sector, there is a paucity of data specifically linking their occupational lead exposure to cardiovascular outcomes like hypertension. Most existing Nigerian studies have focused on general toxicity or haematological markers.

To address this knowledge gap, this study compares battery technicians with local government office workers in Lagos State. Office workers were selected as a comparison group because they share similar socioeconomic backgrounds and urban environmental exposures as the technicians but lack the specific industrial lead exposure associated with battery repair. This comparison allows for a clearer assessment of whether observed BP elevations are specifically associated with the occupational hazards of the trade. While a previous analysis of this cohort examined haematological outcomes, the cardiovascular implications, specifically the relationship between BLL and BP profiles, have not yet been explored. This study aims to assess and compare hypertension and associated risk factors between these two distinct occupational groups.

## Research methods and design

This cross-sectional study was conducted among 150 battery technicians and 150 local government office workers in Lagos State, Nigeria, between December 2016 and July 2017. The study population was restricted to male participants because the battery repair trade in this region is almost exclusively male; excluding the negligible female population ensured cohort homogeneity and avoided gender-based confounding. Battery technicians were selected through a two-stage simple random sampling (balloting) process from registered branches of the Nigeria Association of Battery Technicians. Local government workers were recruited from junior office staff (grade level six and below) to ensure a socioeconomically comparable control group. Inclusion criteria for both groups included at least 1 year of work experience and no history of severe acute illness. This study is part of a larger occupational health survey investigating the multi-systemic effects of lead exposure among workers in Lagos, Nigeria. The dataset was previously utilised to analyse haematological parameters and safety knowledge.^12^ The present analysis represents a distinct, pre-specified secondary objective focusing exclusively on cardiovascular outcomes (BP profiles and hypertension), which were not addressed in the prior publication.

Data on socio-demographics, occupational history, occupational health history and lifestyle habits were collected using a pre-tested, interviewer-administered structured questionnaire. Blood pressure was measured on the right arm in a seated position using an EN-M6 Comfort IT Automatic OMRON monitor. To ensure measurement reliability, each participant rested for 20 min before measurement; three readings were then taken at 5-min intervals, and the average of these readings was used for analysis. Participants’ body weight and height were measured using the OMRON Body Composition Monitor BF511 (models HBF-511-E/HBF-511T-E) and a Leicester Stadiometer (Seca), respectively. Body mass index (BMI) was subsequently calculated as weight in kilograms divided by the square of height in metres (kg/m^2^). For blood lead analysis, 5 mL of venous blood was collected into ethylene diaminetetraacetic acid (EDTA) bottles, which were properly labelled with participant codes corresponding to their questionnaires to ensure anonymity and data integrity.

Blood lead concentrations were determined using atomic absorption spectrophotometry (AAS), a standard and sensitive method for detecting internal lead dose.

Data analysis was performed using Statistical Package for Social Sciences (SPSS) version 20. Bivariate analyses, including chi-square, Mann–Whitney U and Student’s *t*-tests, were applied at a significance level of *p* ≤ 0.05 to compare variables between the two groups. To address potential confounding and determine the independent association between occupational lead exposure and BP, a multivariable regression analysis was performed. This model adjusted for key covariates, including age, BMI, smoking status, alcohol consumption and duration of employment.

### Consent to participate

Written informed consent was obtained from all participants before enrolment in the study. Participants were provided with clear information about the study objectives, procedures, potential risks and benefits, and they were informed of their right to withdraw at any time without penalty. Consent was documented by signed forms, and for respondents with limited literacy, the forms were read aloud in their preferred language before signed or thumbprint consent was taken in the presence of a witness. Confidentiality was ensured by anonymising responses, and data were used strictly for research purposes.

### Ethical considerations

Ethical clearance was obtained from the Health Research Ethics Committee of the Lagos University Teaching Hospital Nigeria (reference number: ADM/DCST/HREC/APP/759). Permission was also obtained from the Lagos State branch of Nigerian Battery Technician Association and the Chairmen of various local government areas. Informed written consent was obtained from the study participants before conducting interviews and tests. This study followed ethical principles specified in the Declaration of Helsinki, such as respect for individuals, beneficence, non-maleficence and fairness. Only anonymised data were utilised for this study.

## Results

A total of 150 battery technicians and 150 local government office workers were successfully interviewed, weighed, and had their height and BP measured, while blood samples were collected for lead analysis, yielding a 91% response rate. Table 1 shows that only 4.7% of battery technicians had completed tertiary education compared to 29.3% of office workers, a statistically significant difference (*p* < 0.001). Income distribution also differed significantly (*p* = 0.021), despite both groups having the same proportion (38%) earning between naira (₦)18 000.00 and ₦ 37 999.00 monthly. More battery technicians (48.0%) had over 20 years’ work experience compared to office workers (25.3%) (*p* < 0.001). Similarly, 71.3% of battery technicians worked more than 8 h daily versus 29.3% of office workers (*p* < 0.001). Table 2 reveals that current alcohol use amongst battery technicians was 48.7% and office workers was 54.7% with statistical significant difference. Blood pressure profiles were broadly similar, but the median SBP was statistically significantly higher among battery technicians (124 mmHg) than office workers (119 mmHg) (*p* = 0.029).

**TABLE 1:** Socio-demographic characteristics of respondents.

Variables	Battery technicians (*n* = 150)			Office workers (*n* = 150)			Test stat (*χ*^2^)	*df*	*p*-value
	*n*	%	Mean ± s.d.	*n*	%	Mean ± s.d.			
**Age group (years)**	-	-	43.85 ± 11.56	-	-	45.12 ± 10.25	8.26	4	0.082
21–30	17	11.3	-	10	6.7	-	-	-	-
31–40	47	31.3	-	38	25.3	-	-	-	-
41–50	49	32.7	-	61	40.7	-	-	-	-
51–60	20	13.3	-	31	20.7	-	-	-	-
> 60	17	11.3	-	10	6.7	-	-	-	-
**Education**	-	-	-	-	-	-	21.53	3	< 0.001**
None	8	5.3	-	4	2.7	-	-	-	-
Primary	72	48.0	-	46	30.7	-	-	-	-
Secondary	63	42.0	-	56	37.3	-	-	-	-
Tertiary	7	4.7	-	44	29.3	-	-	-	-
**Monthly income (naira [₦])**	-	-	-	-	-	-	-	5	0.021*,**
< 18 000.00	54	36.0	-	33	22.0	-	-	-	-
18 000.00 – 37 999.00	57	38.0	-	57	38.0	-	-	-	-
38 000.00 – 57 999.00	32	21.3	-	42	28.0	-	-	-	-
58 000.00 – 77 999.00	3	2.0	-	12	8.0	-	-	-	-
78 000.00 – 97 999.00	2	1.3	-	4	2.7	-	-	-	-
> 98 000.00	2	1.3	-	2	1.3	-	-	-	-
**Years of work**	-	-	-	-	-	-	18.46	2	< 0.001**
0–9	29	19.3	-	53	35.3	-	-	-	-
10–19	49	32.7	-	59	39.3	-	-	-	-
20 and above	72	48.0	-	38	25.3	-	-	-	-
**Daily work hours**	-	-	-	-	-	-	52.92	1	< 0.001**
0–8	43	28.7	-	106	70.7	-	-	-	-
> 8	107	71.3	-	44	29.3	-	-	-	-

*Source*: Adapted from Ozomata EA, Maduafokwa BA, Giwa O, Igwilo UA, Osagiede EF. Assessment of blood lead level and knowledge of health hazards, safety practices and work habits amongst battery technicians in Nigeria. Sci J Public Health. 2025;13(6):338–347. https://doi.org/10.11648/j.sjph.20251306.13, under the CC BY 4.0 license^13^

Note: Bold values indicate level of significance *p*-value less than or equals 0.05.

s.d., standard deviation.

* , Fisher’s exact *p*-value.

** , Statistical significant.

**TABLE 2:** Behavioural and anthropometric risk factors of respondents.

Variable	Battery technicians (*n* = 150)			Office workers (*n* = 150)			*χ* ^2^	*df*	*p*-value
	*n*	%	Mean ± s.d.	*n*	%	Mean ± s.d.			
**Cigarette smoking status**	-	-	-	-	-	-	3.82	2	0.148
Nonsmoker	120	80.0	-	113	75.3	-	-	-	-
Ex-smoker	7	4.7	-	16	10.7	-	-	-	-
Current smoker	23	15.3	-	21	14.0	-	-	-	-
**Alcohol consumption status**	-	-	-	-	-	-	8.00	2	0.012**
Lifetime abstainer	71	47.3	-	52	34.7	-	-	-	-
Former drinker	6	4.0	-	16	10.7	-	-	-	-
Current drinker	73	48.7	-	82	54.7	-	-	-	-
**BMI group**	-	-	-	-	-	-	-	3	0.052*
< 25	111	74.0	-	92	61.3	-	-	-	-
25–29.99	32	21.3	-	45	30.0	-	-	-	-
30–34.99	5	3.2	-	12	8.0	-	-	-	-
35–39.99	2	1.3	-	1	0.7	-	-	-	-
Mean BMI	-	-	22.53 ± 4.30	-	-	24.10 ± 3.95	-	-	-

Note: Bold values indicate level of significance *p*-value less than or equals 0.05.

BMI, body mass index; s.d., standard deviation; *df*, degree of freedom.

* , Fishers exact *p*-value.

** , Statistically significant.

Table 3 shows that diastolic blood pressure (DBP) and grouped BP categories did not differ significantly (*p* > 0.05). Notably, 30.0% of battery technicians had BLL ≥ 5.00 µg/dL, whereas all office workers had BLL < 5.00 µg/dL. Age and years of work were significantly associated with hypertension. Among battery technicians, 23.4% aged 40–59 years had systolic hypertension versus 12.7% under 40 years (*p* = 0.017). Workers with ≥ 11 years’ work duration had higher systolic hypertension (26.1%) than those with fewer years of work (8.6%) (*p* = 0.035). Similar trends were observed among office workers: 23.8% with ≥ 11 years’ experience had systolic hypertension versus 11.4% with less (*p* = 0.050) (Table 4). Diastolic hypertension was most common among battery technicians aged ≥ 60 years (38.9%) compared to 10.9% under 40 years (*p* = 0.029), and more prevalent in those with > 10 years’ experience (24.3% vs. 5.7%; *p* = 0.028). For office workers, 25.0% with > 10 years’ experience had diastolic hypertension compared to 8.6% with less (*p* = 0.008), while 29.5% working > 8 h daily had diastolic hypertension versus 12.3% working less (*p* = 0.011) (Table 5).

**TABLE 3:** Distribution of blood pressure categories and blood lead levels of respondents.

Variable	Battery technicians (*n* = 150)				Office workers (*n* = 150)				Test statistic (*Z*)	*p*-value
	*n*	%	Median	Range	*n*	%	Median	Range		
**Systolic BP (mmHg)‡**	-	-	124.34	90.33–199.00	-	-	119.33	75.33–203.76	−2.180†	0.029**
< 140	117	78.0	-	-	123	82.0	-	-	-	-
140–159.99	25	16.7	-	-	21	14.0	-	-	-	-
160–179.99	5	3.3	-	-	5	3.3	-	-	-	-
≥ 180	3	2.0	-	-	1	0.7	-	-	-	-
**Diastolic BP (mmHg)§**	-	-	79.81	56.33–126.00	-	-	77.08	40.67–142.33	−1.609†	0.108
< 90.00	120	80.0	-	-	124	82.7	-	-	-	-
90–99.99	18	12.0	-	-	21	14.0	-	-	-	-
100–109.99	5	3.3	-	-	3	2.0	-	-	-	-
≥ 110	7	4.7	-	-	2	1.3	-	-	-	-
**BLL groups (µg/dL)**	-	-	-	-	-	-	-	-	161.54	< 0.001**
0–4.99	45	30.0	-	-	150	100.0	-	-	-	-
5–9.99	33	22.0	-	-	0	0.0	-	-	-	-
10–19.99	56	37.3	-	-	0	0.0	-	-	-	-
≥ 20	16	10.7	-	-	0	0.0	-	-	-	-

*Source*: Adapted from Ozomata EA, Ogunnowo B. Assessment of blood lead and haemoglobin levels among battery technicians and local government office workers in South-western Nigeria. Journal of the College of Community Physicians of Sri Lanka. 2025 Jan 29;30(4)

Note: The systolic and diastolic blood pressure is a primary original data compilation which has not been published anywhere else. Bold values indicate level of significance *p*-value less than or equals 0.05.

BP, blood pressure; BLL, blood lead levels.

*, Fisher exact value.

** , Statistically significant.

† , *Z* = Mann–Whitney *U* test;

‡ , *p* = 0.702*;

§ , *p* = 0.314*.

**TABLE 4:** Association between socio-demographics, occupational, behavioural and anthropometric risk factors of participants and their systolic blood pressure.

Variable	Battery technicians (*n* = 150)				Test stat. (*χ*^2^)	*p*-value	Office workers (*n* = 150)				Test stat. (*χ*^2^)	*p*-value
	SBP mmHg < 140 (*n* = 117)		SBP mmHg ≥ 140 (*n* = 33)				SBP mmHg < 140 (*n* = 123)		SBP mmHg ≥ 140 (*n* = 27)			
	*n*	%	*n*	%			*n*	%	*n*	%		
**Age group (years)**	-	-	-	-	8.13	0.017**	-	-	-	-	-	0.202*
< 40	48	87.3	7	12.7	-	-	37	90.2	4	9.8	-	-
40–59	59	76.6	18	23.4	-	-	78	79.6	20	20.4	-	-
≥ 60	10	55.6	8	44.4	-	-	8	72.7	3	27.3	-	-
**Level of education**	-	-	-	-	-	0.375*	-	-	-	-	-	1.000*
No formal	5	62.5	3	37.5	-	-	3	75.0	1	25.0	-	-
At least primary	112	78.9	30	21.1	-	-	120	82.2	26	17.8	-	-
**Monthly income (naira [₦])**	-	-	-	-	1.40	0.237	-	-	-	-	-	0.601*
< 18 000	45	83.3	9	16.7	-	-	25	86.2	4	13.8	-	-
18 000 and above	72	75.0	24	25.0	-	-	98	81.0	23	19.0	-	-
**Years of work**	-	-	-	-	-	0.035*,**	-	-	-	-	3.84	0.050
1–10	32	91.4	3	8.6	-	-	62	88.6	8	11.4	-	-
> 10	85	73.9	30	26.1	-	-	61	76.2	19	23.8	-	-
**Daily work hours**	-	-	-	-	0.04	0.841	-	-	-	-	0.25	0.614
0–8	34	79.1	9	20.9	-	-	88	83.0	18	17.0	-	-
> 8	83	77.6	24	22.4	-	-	35	79.5	9	20.5	-	-
**BMI**	-	-	-	-	2.36	0.124	-	-	-	-	0.06	0.807
< 25	90	81.1	21	18.9	-	-	76	82.6	16	17.4	-	-
25 and above	27	69.2	12	30.8	-	-	47	81.0	11	19.0	-	-
**Smoking status**	-	-	-	-	-	0.669*	-	-	-	-	-	1.000*
Nonsmoker	95	79.2	25	20.8	-	-	93	82.3	20	17.7	-	-
Ex-smoker	5	71.4	2	28.6	-	-	13	81.2	3	18.8	-	-
Current smoker	17	73.9	6	26.1	-	-	17	81.0	4	19.0	-	-
**Alcohol status**	-	-	-	-	-	0.692*	-	-	-	-	-	0.674*
Lifetime abstainer	53	74.6	18	25.4	-	-	44	84.6	8	15.4	-	-
Former drinker	5	83.3	1	16.7	-	-	14	87.5	2	12.5	-	-
Current drinker	59	80.8	14	19.2	-	-	65	79.3	17	20.7	-	-

SBP, systolic blood pressure; BMI, body mass index; stat., statistic.

* , Fishers exact *p*-value.

** , Statistically significant.

**TABLE 5:** Association between socio-demographics, occupational history, behavioural and anthropometric risk factors of participants and their diastolic blood pressure.

Variable	Battery technicians (*n* = 150)				Test stat. (*χ*^2^)	*p*-value	Office workers (*n* = 150)				Test stat. (*χ*^2^)	*p*-value
	DBP mmHg < 90 (*n* = 120)		DBP mmHg ≥ 90 (*n* = 30)				DBP mmHg < 90 (*n* = 124)		DBP mmHg ≥ 90 (*n* = 26)			
	*n*	%	*n*	%			*n*	%	*n*	%		
**Age group (years)**	-	-	-	-	7.06	0.029**	-	-	-	-	3.54	0.176
< 40	49	89.1	6	10.9	-	-	36	87.8	5	12.2	-	-
40–59	60	77.9	17	22.1	-	-	81	82.7	17	17.3	-	-
≥ 60	11	61.1	7	38.9	-	-	7	63.6	4	36.4	-	-
**Level of education**	-	-	-	-	-	0.661*	-	-	-	-	-	0.537*
No formal	6	75.0	2	25.0	-	-	3	75.0	1	25.0	-	-
At least primary	114	80.3	28	19.7	-	-	121	82.9	25	17.1	-	-
**Monthly income (naira [₦])**	-	-	-	-	0.59	0.444	-	-	-	-	0.00	0.988
< 18 000.00	45	83.3	9	16.7	-	-	24	82.8	5	17.2	-	-
18 000.00 and above	75	78.1	21	21.9	-	-	100	82.6	21	17.4	-	-
**Years of work**	-	-	-	-	-	0.028*,**	-	-	-	-	7.03	0.008**
1–10	33	94.3	2	5.7	-	-	64	91.4	6	8.6	-	-
> 10	87	75.7	28	24.3	-	-	60	75.0	20	25.0	-	-
**Daily work hours**	-	-	-	-	0.40	0.527	-	-	-	-	6.48	0.011**
0–8	33	76.7	10	23.3	-	-	93	87.7	13	12.3	-	-
> 8	87	81.3	20	18.7	-	-	31	70.5	13	29.5	-	-
**BMI**	-	-	-	-	0.31	0.577	-	-	-	-	1.70	0.192
< 25	90	81.1	21	18.9	-	-	79	85.9	13	14.1	-	-
25 and above	30	76.9	9	23.1	-	-	45	77.6	13	22.4	-	-
**Smoking status**	-	-	-	-	-	0.056*	-	-	-	-	-	0.937*
Nonsmoker	100	83.3	20	16.7	-	-	93	82.3	20	17.7	-	-
Ex-smoker	6	85.7	1	14.3	-	-	14	85.7	2	14.3	-	-
Current smoker	14	60.9	9	29.1	-	-	17	81.0	4	19.0	-	-
**Alcohol status**	-	-	-	-	-	0.284*	-	-	-	-	-	0.138*
Lifetime abstainer	60	84.5	11	15.5	-	-	46	88.5	6	11.5	-	-
Former drinker	4	66.7	2	33.3	-	-	15	93.8	1	6.2	-	-
Current drinker	56	76.7	17	23.3	-	-	63	76.8	19	23.2	-	-

Note: Bold values indicate level of significance *p*-value less than or equals 0.05.

DBP, diastolic blood pressure; BMI, body mass index; stat., statistic.

* , Fishers exact *p*-value.

** , Statistically significant.

The multivariable logistic regression analysis (Table 6 and Table 7) identifies several critical independent predictors of hypertension within this study population. Advancing age demonstrated a classic dose-response relationship with BP, as participants aged 60 years and above were significantly more likely to present with elevated systolic pressure (adjusted odds ratio [aOR]: 3.92; 95% confidence interval [CI]: 1.52–10.12; *p* = 0.005) and diastolic pressure (aOR: 3.68; 95% CI: 1.34–10.11; *p* = 0.012) compared to those under 40. Occupational longevity emerged as the most consistent work-related predictor, where having more than 10 years of service significantly increased the odds of elevated SBP (aOR: 2.54; 95% CI: 1.18–5.46; *p* = 0.017) and DBP (aOR: 2.86; 95% CI: 1.25–6.54; *p* = 0.013). Furthermore, work intensity was specifically associated with diastolic pressure, as working more than 8 h daily was an independent risk factor for elevated DBP (aOR: 2.12; 95% CI: 1.09–4.13; *p* = 0.027). These findings suggest that the observed cardiovascular risks are primarily driven by cumulative environmental exposure and work duration rather than job category alone, as the technician designation itself was not a significant independent predictor in the adjusted model.

**TABLE 6:** Multivariable logistic regression of factors associated with elevated systolic blood pressure.

Variable	aOR	95% CI	*p*-value
**Occupational group**	-	0.79–2.41	0.258
Office workers (Ref)	1.00	-	-
Battery technicians	1.38	-	-
**Age group (years)**	-	1.14–4.17	0.019*
< 40 (Ref)	1.00	1.52–10.12	0.005*
40–59	2.18	-	-
≥ 60	3.92	-	-
**Years of work**	-	1.18–5.46	0.017*
1–10 years (Ref)	1.00	-	-
> 10 years	2.54	-	-
**BMI**	-	0.68–2.31	0.472
< 25 (Ref)	1.00	-	-
≥ 25	1.25	-	-
**Smoking status**	-	0.55–2.29	0.751
Nonsmoker (Ref)	1.00	-	-
Ever smoker (current/ex)	1.12	-	-

Note: Bold values indicate level of significance *p*-value less than or equals 0.05.

BMI, body mass index; aOR, adjusted odds ratio; CI, confidence interval.

* , Statistically significant.

**TABLE 7:** Multivariable logistic regression of factors associated with elevated diastolic blood pressure.

Variable	aOR	95% CI	*p*-value
**Occupational group**	-	0.82–2.56	0.198
Office workers (Ref)	1.00	-	-
Battery technicians	1.45	-	-
**Age group (years)**	-	1.02–4.08	0.044*
< 40 (Ref)	1.00	1.34–10.11	0.012*
40–59	2.04	-	-
≥ 60	3.68	-	-
**Years of work**	-	1.25–6.54	0.013*
1–10 years (Ref)	1.00	-	-
> 10 years	2.86	-	-
**Daily work hours**	-	1.09–4.13	0.027*
≤ 8 h (Ref)	1.00	-	-
> 8 h	2.12	-	-
**BMI**	-	0.65–2.14	0.584
< 25 (Ref)	1.00	-	-
≥ 25	1.18	-	-

Note: Bold values indicate level of significance *p*-value less than or equals 0.05.

BMI, body mass index; aOR, adjusted odds ratio; CI, confidence interval.

* , Statistically significant.

## Discussion

Most participants were middle-aged and in their productive years, a finding consistent with studies from Iran, Pakistan, Bangladesh and other regions of Nigeria.^10,14,15,16,17^ This confirms that battery repair is a significant source of employment for the Nigerian workforce, emphasising the need for targeted occupational health policies. Battery technicians in this study had lower educational attainment and worked significantly longer daily hours (over 8 h) compared to office workers. This aligns with findings from Ghana and Iran, where informal sector workers often face prolonged hazard exposure because of the absence of formal civil service schedules.^10,17,18^

The prevalence of cigarette smoking (under 20%) was lower in this study than in similar cohorts in Macedonia, Pakistan and Italy^1,6,19^ While smoking is a known contributor to lead manifestations and cardiovascular risk,^9,20,21^ the lack of a significant difference between our two study groups suggests that smoking was not a primary driver of the observed BP differences. Conversely, alcohol consumption was prevalent in both groups, which may exacerbate lead toxicity by altering lead absorption and distribution.^16,22,23^ The mean BMI of battery technicians (22.53 kg/m^2^ ± 4.30 kg/m^2^) was significantly lower than that of office workers (24.10 kg/m^2^ ± 3.95 kg/m^2^), although both remained within the normal range. The higher BMI among office workers likely reflects the sedentary nature of civil service work. Consistent with several international studies, BMI was not significantly associated with BLL in this specific cohort, although evidence on this relationship remains inconsistent globally.^24,25^

A key finding was that median SBP was significantly higher in battery technicians than in office workers (*p* = 0.029). This association between occupational group and SBP is consistent with findings in Italy, Taiwan and India, where lead exposure is hypothesised to affect the renin–angiotensin system and renal function.^5,7,9^ Notably, differences in DBP were not significant, which mirrors results from Iran and India suggesting that lead’s impact on BP may be primarily systolic.^8,10^ Among technicians, SBP was significantly associated with age and years of work. While age is a non-modifiable risk factor, the association with ‘years of work’ may reflect cumulative occupational exposure.^7^ Similarly, DBP in technicians was linked to age, while in office workers, it was associated with work duration and daily hours, factors potentially influenced by sedentary stress and lifestyle habits.^7,24^

The significant association between over 10 years of service and both SBP and DBP suggests that the cardiovascular impact of this environment, whether through lead exposure in technicians or prolonged sedentary stress in office workers, is cumulative. This underscores the importance of longitudinal monitoring rather than just cross-sectional screening. The strong dose-response relationship with age further complicates this, as it suggests that the natural biological decline in vascular elasticity is exacerbated by these occupational durations. Another significant finding was the association between work intensity (over 8 h daily) and elevated DBP. This suggests that the ‘pressure’ of the workday, be it physical or psychological, could influence peripheral arterial resistance. Because the job category itself lost significance in the adjusted model, these results imply that an office worker with long hours and a decade of service may face similar diastolic risks as a technician, highlighting that work-related hypertension in this cohort is a multi-faceted issue driven by how long and how hard one works, rather than just where they work.

### Limitations

The cross-sectional design of this study precludes the determination of a causal relationship between lead exposure and hypertension. While multivariable logistic regression was employed to control for known confounders, such as age, BMI and work duration, the potential for residual confounding from unmeasured variables remains. The study may also be subject to selection bias, as participants were drawn from specific occupational hubs, which may not fully represent the diversity of the informal battery repair sector. Furthermore, the reliance on self-reported data for lifestyle factors may introduce recall bias. Regarding lead assessment, the use of a single blood sample provides a snapshot of current exposure rather than a lifetime cumulative dose, which is better captured through bone lead measurements. Finally, while this study utilises a dataset from which haematological findings have been previously published, the clinical focus on hypertension is novel for this cohort. We have ensured clear disclosure to avoid redundant reporting, emphasising that the cardiovascular risks identified here provide a unique contribution to the understanding of lead toxicity in Nigeria’s informal sector.

## Conclusion and recommendations

This study demonstrates that hypertension in the study population is independently driven by advancing age, occupational longevity and work intensity rather than job category alone. Multivariable analysis reveals that having over 10 years of service and working more than 8 h daily are significant predictors of elevated BP, suggesting that the cardiovascular risks are a result of cumulative environmental exposure and prolonged occupational stress. Specifically, the strong association between elevated BLL and increased BP among battery technicians underscores the critical health burden within the informal sector. To mitigate these risks, workplace processes must be modified to minimise lead fume exposure, such as enclosing batteries during repair. It is essential to implement regular occupational safety training and ensure the consistent use of personal protective equipment, including lead-resistant aprons, gloves and face masks. Furthermore, improving workplace hygiene with accessible running water and hand-washing facilities is vital to reducing lead ingestion. Occupational health authorities should institute routine biological monitoring of BLL alongside periodic medical screenings for early detection of hypertension. Finally, policymakers must strengthen the regulation and enforcement of health standards within the informal sector to protect these vulnerable workforces from long-term cardiovascular morbidity.
